# Hamaker Constants of van der Waals Materials under Clean‐Interface Conditions

**DOI:** 10.1002/advs.76609

**Published:** 2026-07-17

**Authors:** Daniel Gallego‐Fuente, Jaime Colchero, Julio Gomez‐Herrero, Pablo Ares

**Affiliations:** ^1^ Departamento de Física de la Materia Condensada Universidad Autónoma de Madrid Madrid Spain; ^2^ Condensed Matter Physics Center (IFIMAC) Universidad Autónoma de Madrid Madrid Spain; ^3^ Instituto Nicolás Cabrera Universidad Autónoma de Madrid Madrid Spain; ^4^ Optics and Nanophysics Research Center Universidad de Murcia Murcia Spain

**Keywords:** 2D materials, atomic force microscopy, graphene, Hamaker, MoS_2_, van der Waals interactions

## Abstract

Adhesion stabilizes van der Waals materials and heterostructures, yet reported interaction strengths show a wide dispersion that hinders a consistent link between microscopic dispersive forces and macroscopic observables. Here, we determine clean‐interface adhesion by measuring tip‐sample interactions in ultrahigh vacuum using calibrated AFM probes of different radii, in situ thermal cleaning, and realistic contact distances. This approach isolates the dispersive interaction of clean interfaces and allows us to extract mixed Hamaker constants for SiO_2_ interacting with few‐layer graphene (12 × 10^−20^ J), MoS_2_ (13 × 10^−20^ J), and SiO_2_ (6.1 × 10^−20^ J). Agreement between static adhesion spectroscopy and noncontact frequency‐modulation AFM (FM‐AFM) provides an internal consistency check of the interaction scale. Controlled humidity cycles further show that even very dry ambient conditions already enhance adhesion, while higher humidity produces a strong reversible increase associated with capillary forces, explaining one major contribution to the large spread of commonly reported values. These results establish clean‐interface experimental benchmark values for dispersive interactions in representative layered materials, enabling quantitative comparison with dielectric‐response and Lifshitz‐based models that relate Hamaker constants to binding energy and macroscopic properties of van der Waals crystals.

## Graphical abstract

Clean‐interface adhesion in representative van der Waals materials is quantified by Ultra High Vacuum (UHV) Atomic Force Microscopy (AFM) with in situ cleaning and calibrated probes. Benchmark Hamaker constants for graphene, MoS_2_, and SiO_2_ are established, while humidity is shown to reversibly amplify adhesion, explaining much of the scatter in reported values.

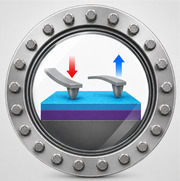

## 1 Introduction

The assembly of van der Waals (vdW) heterostructures has enabled materials design with atomic precision, but their structural integrity relies on weak interfacial vdW forces [1]. Quantifying adhesion is therefore essential for device stability, deterministic transfer [2], electronic integration [3], and structural superlubricity [4]. Yet, reported experimental values remain widely scattered. While the blister test provides an important benchmark [5], nanoscale measurements with atomic force microscopy (AFM) are often affected by surface roughness, transfer residues, and, most importantly, environmental adsorbates [6, 7]. vdW materials are sensitive to airborne contamination, which rapidly modifies their apparent wetting and interfacial properties [8, 9, 10]. In particular, graphite and few‐layer graphene (FLG) combine broad relevance with an especially dispersed experimental literature [1, 4, 5, 11, 12, 13, 14]. They provide an ideal model system to examine this problem, while MoS_2_ offers a complementary semiconducting reference. Reliable experimental Hamaker constants are also needed as inputs for quantitative adhesion models in realistic nanoscale contacts, where geometry, roughness, and environmental adsorbates can strongly modify vdW forces [15, 16, 17]. This makes clean and calibrated measurements essential for benchmarking theory, comparing material systems, and connecting AFM‐based vdW quantification with transfer, particle‐removal, and nanoscale‐contact problems.

More in detail, a central challenge is that ambient interfaces do not reflect the clean‐interface dispersive interaction of 2D materials. Capillary condensation and adsorbed layers can mask the underlying vdW forces and, in particular, distort AFM measurements [18, 19, 20, 21, 22]. In AFM operated under ambient conditions, this masking often appears through the formation of water menisci that modify the underlying vdW interaction, which is typically quantified through the Hamaker constant. Beyond environmental effects, the determination of this constant is also affected by methodological differences. As a result, even values obtained under nominally similar conditions can still differ substantially across studies.

Here we determine clean‐interface adhesion by combining ultrahigh vacuum (UHV) with in situ thermal cleaning, calibrated large‐radius probes that preserve the elastic regime, realistic contact distances, and cross‐checks with frequency‐modulation AFM (FM‐AFM), which relies on different underlying assumptions. This approach enables us to distinguish the dispersive interaction of clean interfaces from environmental and methodological artefacts when determining Hamaker constants.

## 2 Methods

**FIGURE 1:**
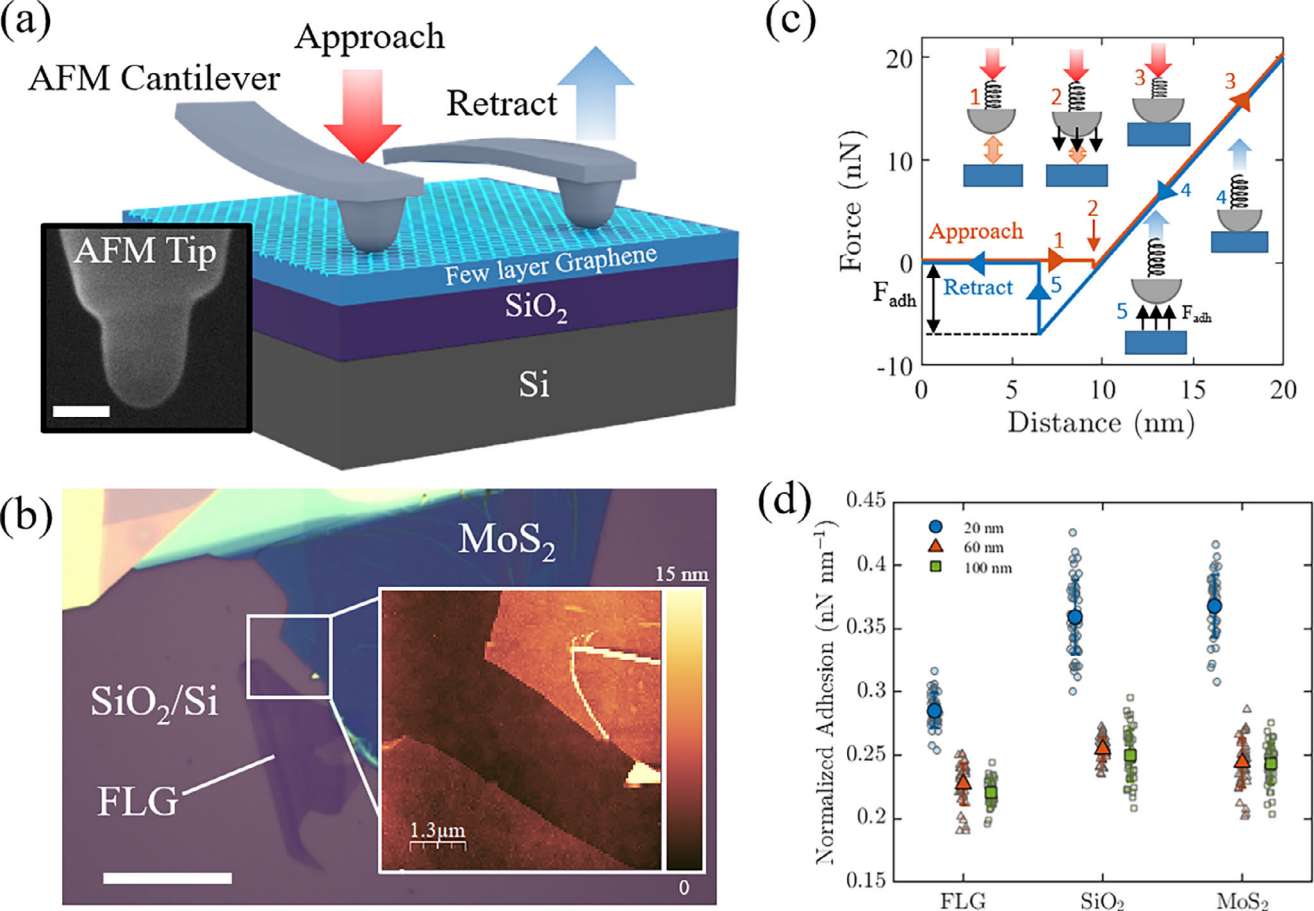
(a) Schematics of the AFM adhesion measurement. Inset: SEM micrograph of a used silicon probe (scale bar, 100 nm). (b) Optical micrograph of mechanically exfoliated FLG and dry‐transferred MoS_2_ on SiO_2_ (scale bar, 10 µm). Inset: AFM topography of the same region. (c) Schematic force vs. distance curve corresponding to the method shown in (a). During approach, the tip evolves from the non‐interacting regime (1) through the snap‐in instability (2) into repulsive contact (3). During retraction, adhesive forces keep the tip in contact (4) until the elastic restoring force overcomes the attraction at the jump‐off instability (5). *F_adh_* is the maximum attractive force recorded at jump‐off, as indicated on the Y‐axis. (d) Adhesion measured in UHV, normalized by the tip radius, for probes with *R ≈* 20, 60, and 100 nm. The 60 and 100 nm probes define the converged large‐radius regime used for quantitative Hamaker extraction, whereas the 20 nm data are retained as a small‐radius control.

We carried out the experiments in a homemade beam‐deflection AFM controlled by WSxM [23, 24] inside an ultrahigh vacuum chamber with base pressure of 10^−9^ mbar. We used cantilevers with nominal tip radii of 20, 60, and 100 nm (Section S1). For each nominal radius, the same probe was used to measure the FLG, MoS_2_, and SiO_2_ regions, and its radius was determined by post‐measurement Scanning Electron Microscopy (SEM). The samples consisted of few‐layer flakes on SiO_2_/Si substrates. FLG was prepared by direct micromechanical exfoliation onto SiO_2_/Si, whereas MoS_2_ flakes were exfoliated onto a polydimethylsiloxane (PDMS) stamp and transferred by deterministic dry transfer on the same substrate [2], close to the FLG flake. Figure 1a,b illustrate the experimental method and the sample region of interest.

Samples were annealed in situ in UHV at 200 °C for 2 h to remove physisorbed water and volatile contaminants while preserving structural integrity [8, 25, 26, 27, 28, 29]. Before/after annealing force spectroscopy confirmed that this procedure reduces and stabilizes the measured adhesion (Figure S2). Silicon AFM probes with 3 N/m nominal stiffness were cleaned first *ex situ* by ozone treatment and then in situ by oxygen plasma to remove organic residues without degrading the tip apex [30, 31, 32]. This treatment also enhances the native oxide layer of the tip.

Accurate force quantification relied on Sader spring‐constant calibration in air [33], and SEM determination of the tip radius *R* [Figure 1a, inset]. Static force spectroscopy was used to extract the adhesion force *F_adh_* from the jump‐off instability during retraction, as illustrated in Figure 1c. Because pull‐off force scales as *F_adh_ ∝ Rγ*, the normalized quantity *F_adh_*/*R* is proportional to the effective work of adhesion *γ* and provides a convenient way to test radius scaling. Finally, we complement these measurements with noncontact FM‐AFM in UHV [34, 35, 36], which probes the attractive regime without mechanical contact and follows a different analysis route based on force reconstruction and long‐range tail fitting (Section S2).

## 3 Results and Discussion

### 3.1 Contact Mechanics and Geometric Consistency

Reliable quantification of the Hamaker constant requires a clean contact regime with reproducible radius scaling. As shown in Figure 1d and summarized statistically in Table S2, the normalized adhesion force *F_adh_/R* converges to a stable value for the larger tip radii (*R ≈* 60 and 100 nm), consistent with the linear scaling with tip radius expected for an elastic contact [37]. In contrast, the sharpest tips (*R ≈* 20 nm) deviate systematically from this plateau, yielding higher normalized adhesion values, possibly due to the increased sensitivity of small‐radius contacts to local stress, apex geometry, and radius‐/stiffness‐dependent adhesive effects in thin supported systems [38, 39, 40]. To discern the adhesion model suitable to describe our measurements, we use the dimensionless Tabor parameter *µ* that was estimated from the measured adhesion values of our probes:

(1) $$\mu ={\left(\frac{16\;R\;\gamma^{2}}{9\;K^{2}z_{0}^{3}}\right)}^{\frac{1}{3}}$$

where *K* is the reduced elastic modulus and *z_0_* is the effective contact distance (see Section S3). The Tabor parameter compares the range of adhesive forces with the elastic deformation scale of the contact. It is commonly used to distinguish between two limiting adhesion regimes: compliant Johnson‐Kendall‐Roberts (JKR)‐type contacts, where elastic deformation and adhesive necking are significant; and low‐Tabor rigid‐body contacts, Bradley/Derjaguin‐Muller‐Toporov (DMT), where the solids remain nearly undeformed during pull‐off. The resulting values for our experiments fall typically around *µ ≈* 0.1– 0.2 (see Table S1). These values place our measurements on the low‐Tabor, rigid‐body side of this transition, where adhesive necking is expected to remain small and the pull‐off force approaches the Bradley/Derjaguin limit [41, 42, 43, 44, 45].

We therefore use the values obtained with the 60 and 100 nm probes as the clean‐interface adhesive baseline for quantitative Hamaker constant extraction and quantify them using the contact model described in Section S3, resulting in 12 × 10^−20^ J for FLG‐SiO_2_, 13 × 10^−20^ J for MoS_2_‐SiO_2_ and 6.1 × 10^−20^ J for SiO_2_‐SiO_2_. All Hamaker constants discussed below are taken from this converged large‐radius regime. The 20 nm data are retained as a small‐radius control, but are not used for quantitative extraction because they do not fall on the converged *F_adh_*/*R* baseline defined by the larger‐radius probes.

### 3.2 Clean‐Interface Adhesion and Hamaker Constant Determination

Working in UHV ensures a clean tip‐sample gap that allows us to determine the clean‐interface adhesion force, minimizing contributions from adsorbates and capillary forces. In this low‐Tabor Bradley/rigid‐body pull‐off regime, the adhesion force can be mapped onto the Hamaker constant *A_12_* of the clean tip‐sample system through Equation (2) (Section S3 for details).

(2) $$A_{12}=\frac{6F_{adh}z_{0}^{2}}{R}$$

where *F_adh_* is the adhesion force obtained as explained in Figure 1, *R* is the tip radius as determined by SEM, and *z_0_* is the effective short‐range separation used to relate the measured adhesion to the Hamaker constant. The subscript *1* refers to the tip material, and *2* to sample material. We intentionally used few‐layer flakes instead of mono‐, bi‐, or trilayers to reduce substrate‐induced contributions to the measured surface interaction, with the lower layers screening the underlying SiO_2_ dispersive force. This choice is particularly relevant for supported graphene, where AFM studies show that layer thickness and substrate‐mediated vdW interactions can affect tip‐graphene adhesion [11, 46].

**FIGURE 2:**
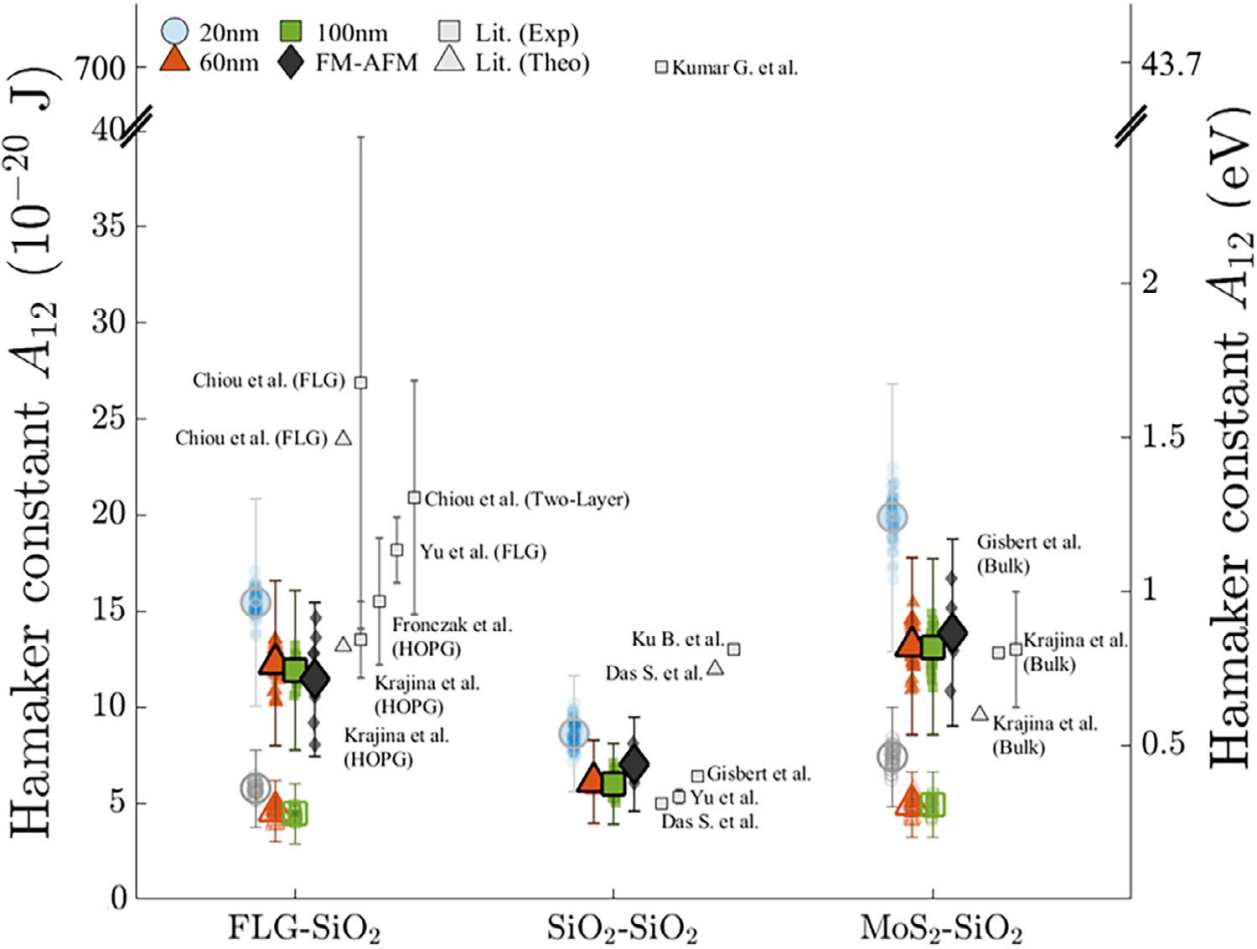
Hamaker constants (*A_12_*) measured in UHV for FLG‐SiO_2_, SiO_2_‐SiO_2_, and MoS_2_‐SiO_2_ (large colored filled symbols), compared with experimental and theoretical literature values (small open symbols). Error bars include conversion and calibration uncertainties. Conversions use *z_0_* = 0.3 nm for FLG and MoS_2_, and *z_0_* = 0.2 nm for SiO_2_. Large light colored open symbols show the FLG and MoS_2_ values obtained with *z_0_* = 0.2 nm. Noncontact FM‐AFM results (large filled rhombs) provide a complementary UHV consistency check based on force reconstruction and long‐range tail fitting. The 20 nm tip results are retained as a small‐radius control. Literature values are taken for graphite [11, 12, 13, 14], MoS_2_ [12, 15], and SiO_2_ [6, 11, 15, 16, 50].

Figure 2 compares our UHV *A_12_* values with available experimental benchmarks and recent theoretical estimates for graphite, MoS_2_, and SiO_2_. The graphene/graphite system provides the most informative comparison not only because its AFM literature is extensive, but also because it exhibits by far the widest reported spread, making it an especially sensitive testbed for environmental and methodological effects [11, 12, 13, 14]. In this system, our FLG values fall systematically at the lower edge of the reported range, consistent with a clean‐interface limit in which the measured adhesion is not inflated by ambient contributions. For MoS_2_, our results agree in magnitude with two independent AFM approaches based on different observables and analysis routes [12, 15]. The SiO_2_ reference also falls within the lower part of the published range, providing an internal baseline for the absolute scale of the interaction strengths discussed here and helping contextualize the values obtained for the 2D crystals [12, 47, 48].

The dispersion in the literature values seen in Figure 2 does not arise from a single source, but from a combination of uncontrolled environmental conditions and heterogeneous analysis conventions. Measurements performed outside UHV are particularly sensitive to airborne contamination, adsorbates, and interfacial water, which can introduce spurious contributions and inflate the apparent interaction [25, 47, 49]. This sensitivity is especially pronounced for graphite but also affects other surfaces such as oxides, helping to explain why several reported SiO_2_ values exceed our UHV reference under less controlled conditions [25, 48, 50]. Additional spread arises because the adhesion‐to‐Hamaker conversion is not implemented uniformly across the literature, with different AFM approaches relying on different observables, reconstruction procedures, force models, and geometric assumptions [14, 15, 16, 17, 51]. In our case, the agreement between static adhesion spectroscopy and noncontact FM‐AFM provides a complementary consistency check of the interaction scale. The FM‐AFM analysis avoids the pull‐off contact distance used in Equation (2), but it still depends on the absolute tip‐sample separation, the force‐reconstruction procedure, the fitting window, and the effective tip geometry [34, 35].

The contact distance *z_0_* in Equation (2) is an important source of systematic variation in any adhesion‐based extraction of a Hamaker constant [47, 52]. This effective distance also reflects the short‐range repulsive interactions that become relevant at pull‐off and can influence AFM‐based Hamaker estimates [53]. We therefore treat *z_0_* as an explicit effective parameter. Reported values range from hard‐core distances around *z_0_ ≈* 0.165 nm [12, 13, 47, 50] to larger effective separations of *z_0_ ≈* 0.3–0.4 nm adopted to represent realistic scenarios [6, 16]. For graphene on SiO_2_, interface studies place the vdW stabilization distance at about 3 Å, supporting *z_0_ =* 0.3 nm as a realistic scale [54]. Accordingly, Figure 2 uses *z_0_ =* 0.3 nm for FLG and MoS_2_, for which a comparable effective separation is expected. We also show the values obtained with *z_0_ =* 0.2 nm (symbols at the bottom in Figure 2), in line with values commonly used in the literature, which illustrates the strong sensitivity to this assumption. For the SiO_2_‐SiO_2_ reference, where both tip and sample share the same material (as discussed previously), we retain *z_0_ =* 0.2 nm as a conservative choice and propagate an estimated ±25% systematic uncertainty into the final values, as reflected in the error bars of Figure 2 (see Supporting Information) [48].

Importantly, methods that do not prescribe a pull‐off contact distance are not free of uncertainty, but shift it to force reconstruction, calibration, separation definition, or multi‐parameter fitting. Static adhesion spectroscopy instead provides a direct pull‐off measurement, with the contact‐distance convention made explicit through *z_0_*. We therefore complement static adhesion spectroscopy with noncontact FM‐AFM in the attractive regime. The quantitative agreement between both UHV methods provides a consistency check of the interaction scale reported here, rather than a fully model‐independent validation (see Supporting Information) [34, 35, 36].

Our adhesion spectroscopy directly yields the mixed Hamaker constant *A_12_*, the most accessible experimental quantity [5, 47, 52]. Beyond these mixed interactions, comparison with theory is often formulated in terms of the self‐interaction constant *A_11_*, which captures the macroscopic polarizability of a given material. We infer this magnitude using the standard geometric‐mean approximation of Equation (3) [47, 52], providing a compact route to compare our experimental Hamaker constant determination with state‐of‐the‐art theoretical predictions for the self‐interaction of the three probed materials. For this conversion, we consider the *A_11_* of the tip as being equal to the one of SiO_2_, consistent with the silica‐terminated nature of the cleaned probe apex (discussed previously). Therefore, this quantity was directly obtained in the SiO_2_‐SiO_2_ measurements (see Table 1).

(3) $$A_{12}\approx \sqrt{{A_{11}}^{tip}{A_{11}}^{sample}}$$

**TABLE 1:** Inferred *A_11_* values compared with Lifshitz‐based estimates.

Material	Inferred *A_11_* (×10^−20^ J)	Lifshitz *A_11_* [55] (×10^−20^ J)
Graphite	24 ± 13	23.95
SiO_2_	6.1 ± 1.5	7.85
MoS_2_	29 ± 16	31.24

We compare these inferred *A_11_* values with recent state‐of‐the‐art self‐Hamaker constants obtained from dielectric‐response (Lifshitz theory) analysis for graphite, MoS_2_, and SiO_2_ [55]. As summarized in Table 1, our inferred values for graphite and MoS_2_ agree well with these theoretical estimates, while SiO_2_ remains close to the expected value. Although all back‐inferred constants carry propagated experimental uncertainty, their overall agreement with Lifshitz‐based benchmarks clearly supports our approximations and obtained values.

### 3.3 Environmental Artifacts and Reversibility

**FIGURE 3:**
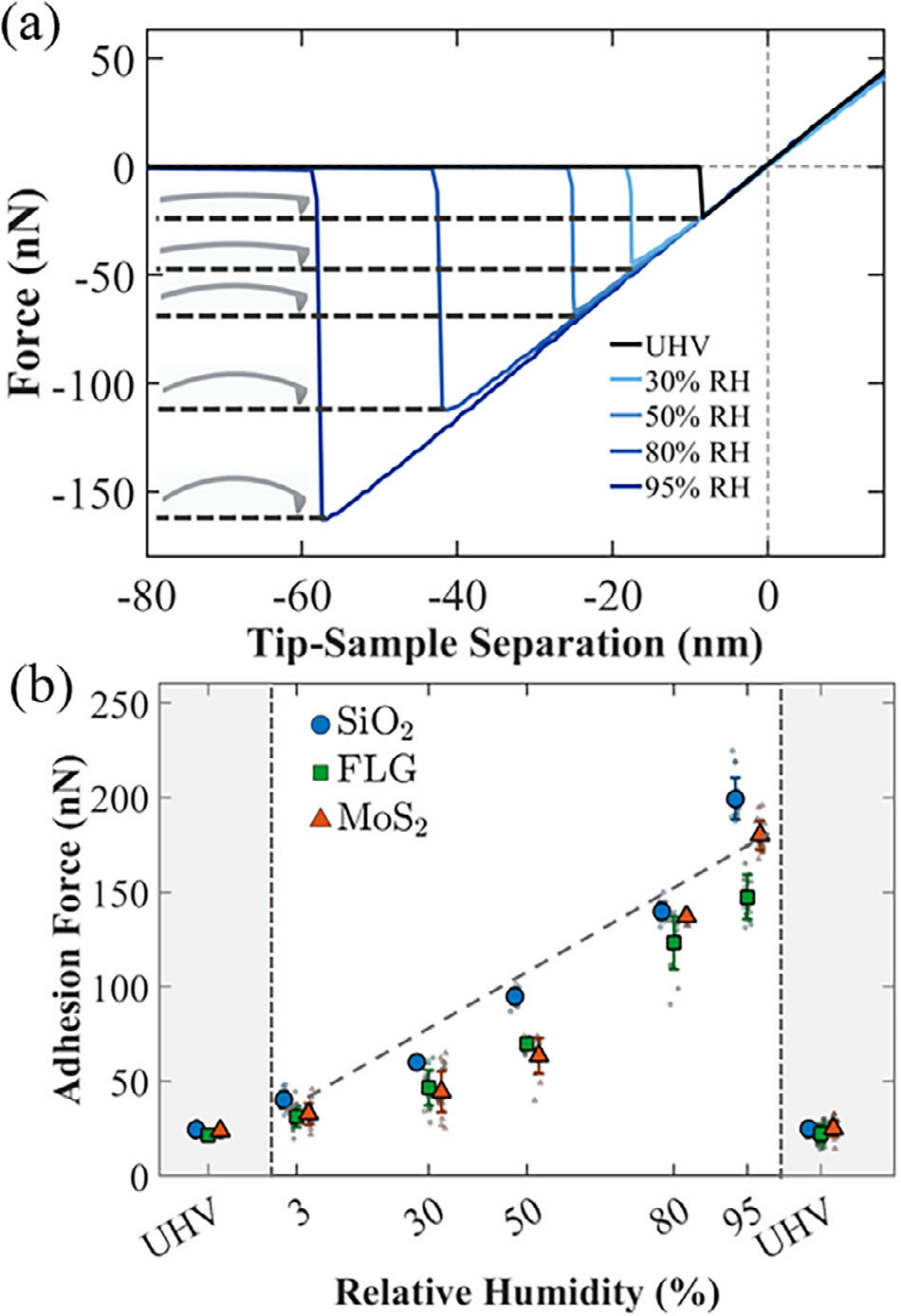
(a) Force‐distance retraction curves for FLG and a *R ≈* 100 nm tip. UHV measurements (black) exhibit low adhesion, while ambient air (blue) reveals an increase of up to one order of magnitude, ascribed to the water meniscus and its humidity dependence. Schematic drawings of the increasing cantilever bending are pictured on the left of each curve for illustrative purposes. (b) Evolution of the adhesion force through the experimental cycle (UHV → Air → UHV), demonstrating recovery of the clean‐interface baseline after thermal cleaning. The dashed line is just a guide for the eye.

Finally, to illustrate how environmental factors can drive the broad dispersion of literature values, we examine the effect of humidity on the measured adhesion. Whereas UHV measurements show the short‐range detachment expected for solid‐solid contact, measurements in ambient air display a pronounced increase in the pull‐off distance, as shown in Figure 3a. This increase reflects the formation of tip‐sample liquid menisci, marking the transition from a regime dominated by van der Waals interactions [47] to one dominated by capillary forces [18, 52, 56, 57].

As shown in Figure 3, meniscus formation increases the measured adhesion by up to one order of magnitude relative to the UHV baseline, consistent with the humidity dependence of water meniscus energy [58] and the thermodynamics of water condensation [59]. This is particularly relevant for graphene surfaces where airborne contaminants and water adlayers readily adsorb [8, 9, 60]. Figure 3b shows the evolution of the adhesion throughout a complete environmental cycle: starting in UHV, increasing the relative humidity up to 95%, and then returning to UHV along with in situ sample cleaning. The strong adhesion enhancement observed in varying ambient conditions fully disappears after reintroduction into UHV, demonstrating that the effect is reversible and excluding permanent chemical aging or degradation. Instead, it shows that the ambient measurement is dominated by removable adsorbates [19]. Notably, even at 3% relative humidity the adhesion already exceeds the UHV baseline by a factor of 1.5 and shows a broader spread, indicating that very dry ambient conditions do not reproduce the UHV clean‐interface baseline. Under typical ambient humidity, the deviation becomes even more pronounced. This controlled reversibility contrasts with the hysteresis and drift often encountered in ambient measurements, where fluctuating adsorbate layers and capillary necks hinder access to the clean‐interface interaction. Together, these results show that strict environmental control is essential to access clean‐interface adhesion and reliable Hamaker constants.

## 4 Conclusions

We have determined Hamaker constants under clean‐interface conditions for few‐layer graphene, MoS_2_, and SiO_2_ by directly measuring adhesion in UHV with in situ cleaning, calibrated large‐radius probes, and realistic contact distances. Agreement with UHV FM‐AFM measurements and with Lifshitz‐based predictions indicates that these values provide a reliable experimental scale for the clean‐interface dispersive interaction of the investigated interfaces. Controlled humidity cycles further show that capillary bridging and adsorbates reversibly enhance adhesion under ambient conditions, identifying one important contribution to the frequent overestimation and scatter seen in reported values. Our results therefore help to establish the experimental conditions required for reliable characterization of van der Waals adhesion and Hamaker constant determination, clarifying the origin of the large dispersion in the literature and providing experimentally anchored parameters for predictive adhesion models in realistic nanoscale contacts.

## Conflicts of Interest

The authors declare no conflicts of interest.

## Supporting information




**Supporting File**: advs76609‐sup‐0001‐SuppMat.docx.

## Data Availability

The data that support the findings of this article are openly available in Zenodo at https://zenodo.org/, ref. [61].
