# Supplementary material for: Hamaker Constants of van der Waals Materials under Clean‐Interface Conditions
Source: Adv Sci (Weinh). 2026 Jul 17:e76609. Online ahead of print. doi: 10.1002/advs.76609 (PMC13379217; doi:10.1002/advs.76609)
Supplement: Supplementary file 1 — Supporting File: advs76609‐sup‐0001‐SuppMat.docx. [file ADVS-9999-e76609-s001.docx]

**Supporting Information for Hamaker Constants of van der Waals Materials under Clean-Interface Conditions**

Daniel Gallego-Fuente, Jaime Colchero, Julio Gomez-Herrero*, Pablo Ares*

D. Gallego-Fuente, J. Gomez-Herrero, P. Ares

Departamento de Física de la Materia Condensada. Universidad Autónoma de Madrid, Madrid, E-28049, Spain

D. Gallego-Fuente, J. Gomez-Herrero, P. Ares

Condensed Matter Physics Center (IFIMAC), Universidad Autónoma de Madrid, E-28049, Madrid, Spain

E-mails: julio.gomez@uam.es; pablo.ares@uam.es

D. Gallego-Fuente, J. Gomez-Herrero, P. Ares

Instituto Nicolás Cabrera, Universidad Autónoma de Madrid, E-28049, Madrid, Spain

J. Colchero

Optics and Nanophysics Research Center, Universidad de Murcia, 30100, Murcia, Spain

**S1. EXPERIMENTAL METHODS**

**A. Sample Preparation**

All samples were fabricated on commercially available silicon wafers (MicroChemicals GmbH) with a
290 nm thermally grown SiO_2_ layer. This oxide thickness was selected to optimize the optical contrast for the identification of few-layer flakes. Prior to material deposition, the substrates were ultrasonically cleaned in acetone and isopropanol (IPA) for 10 minutes each, followed by drying with a stream of pure nitrogen gas. To ensure a pristine surface free of organic residues, the substrates underwent an oxygen plasma treatment for 15 minutes immediately before the exfoliation or transfer steps.

Few-layer graphene (FLG) samples were prepared using the standard micromechanical exfoliation method. Natural graphite flakes (NGS Naturgraphit GmbH) were peeled repeatedly using adhesive tape to thin the crystals and then pressed onto the cleaned SiO_2_/Si substrates. After deposition, optical microscopy (Zeiss Imager.A2m) was used to identify few-layer flakes based on their optical contrast. Few-layer MoS_2_ flakes were fabricated using a deterministic dry transfer technique [1]. Bulk MoS_2_ crystals (HQ Graphene) were first micromechanically exfoliated onto a viscoelastic polydimethylsiloxane (PDMS) stamp (Gel-Pak Standard Silicone Gel Film). The stamp was inspected under an optical microscope to select flakes of appropriate thickness and lateral dimensions. Using a micromanipulator, the selected flakes were aligned over the target SiO_2_/Si substrate and brought into contact, after which the PDMS stamp was peeled off to transfer the MoS_2_ flakes.

To remove surface contamination (water adlayers and airborne hydrocarbons) adsorbed during air exposure [2], samples were introduced into the Ultra-High Vacuum (UHV) chamber immediately after fabrication. Once in UHV (base pressure 10^-9^ mbar), the samples underwent a specific in-situ thermal annealing cycle, with the annealing stage shown in Fig. S1(a). To preserve the structural integrity of the 2D crystals and avoid the formation of thermal stress-induced ripples [3], we employed a slow ramping protocol. The temperature was increased from ambient to 200 °C over a period of 2 hours, maintained at 200 °C for 2 hours, and subsequently cooled back to ambient temperature over another 2-hour period. This protocol is sufficient to desorb physisorbed water and volatile organics [4, 5] while avoiding sulfur desorption in MoS_2_ [6] or the aggregation of amorphous carbon residues [7]. Force spectroscopy measurements commenced shortly after the samples had completely thermalized to room temperature.


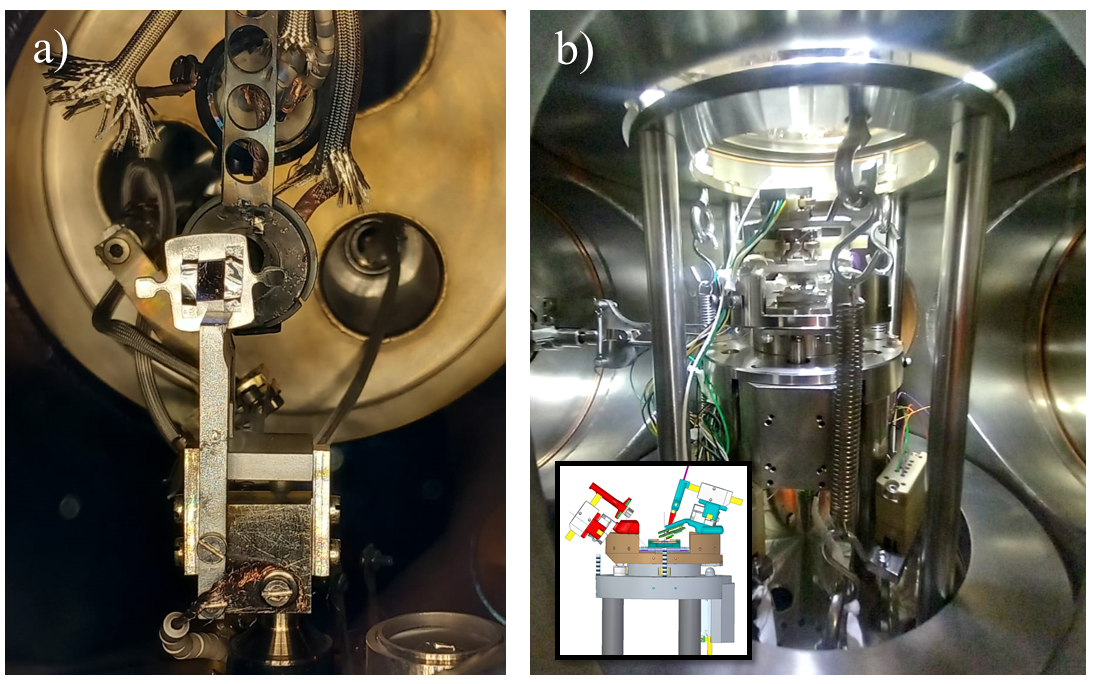


**FIG S1.** Experimental setup inside the ultra-high vacuum (UHV) system. (a) Close-up of the sample annealing stage used for in-situ thermal cleaning. (b) Photograph of the home-built beam deflection AFM head suspended by vibration isolation springs. Inset showing side-view schematic of AFM head, optical path visible.

The effect of the cleaning protocol was checked by comparing normalized adhesion measurements before and after annealing. Unannealed samples showed higher and more dispersed *F_adh_/R* values, whereas annealed samples converged to the lower and more reproducible baseline used for Hamaker constant extraction (Fig. S2).


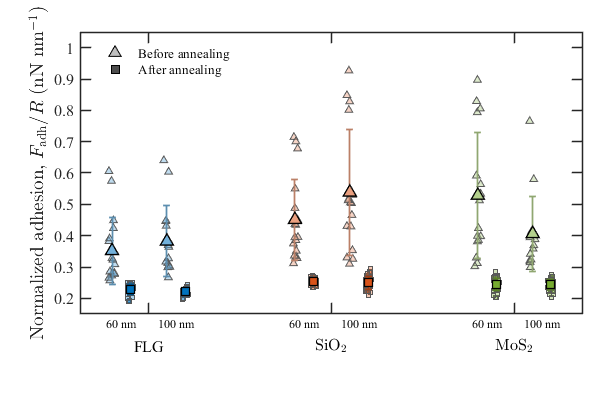


**FIG S2.** Effect of in-situ thermal cleaning on normalized adhesion. Normalized adhesion values *F_adh_/R* measured before and after UHV annealing. Unannealed samples show higher and more dispersed adhesion, whereas annealed samples converge to the low-adhesion baseline used for Hamaker constant extraction.

**B. AFM Instrumentation and Probe Calibration**

We carried out experiments in a homemade system equipped with a beam-deflection atomic force microscope (AFM) controlled by WSxM [8, 9] inside an Ultra-High Vacuum chamber (base pressure
~10^−9^ mbar). The setup comprises a preparation chamber coupled to the main analysis chamber. For static force spectroscopy, we employed silicon cantilevers with nominal tip radii of 20, 60, and 100 nm and
*C =* 3.0 N/m (Team Nanotec; Models HSC 20, HSC 60, and HSC 100, respectively). To cross-validate our results, we utilized PPP-NCLR probes (Nanosensors) optimized for frequency modulation (FM-AFM).

To eliminate organic contaminants on the probes, we applied a two-step cleaning protocol. First, we subjected the probes to a 10-minute ultraviolet-ozone treatment in ambient air using an Ossila UV Ozone Cleaner (Ossila Ltd., UK). Then, we immediately introduced the probes into the UHV system and performed an in-situ oxygen plasma cleaning for an additional 10 minutes [10].

For quantitative analysis, we calibrated the normal spring constants using the Sader method [11] in ambient conditions both before and after UHV measurements. Crucially, as the tip radius (R) governs the contact mechanics models, we did not rely on nominal manufacturer specifications. Instead, we characterized the specific geometry of each tip via high-resolution Scanning Electron Microscopy (SEM, FEI Verios 460) post-measurement. We used these experimentally determined radii for all subsequent data normalization and Hamaker constant calculations. Figure S3 presents SEM micrographs of the specific probes used for force spectroscopy.


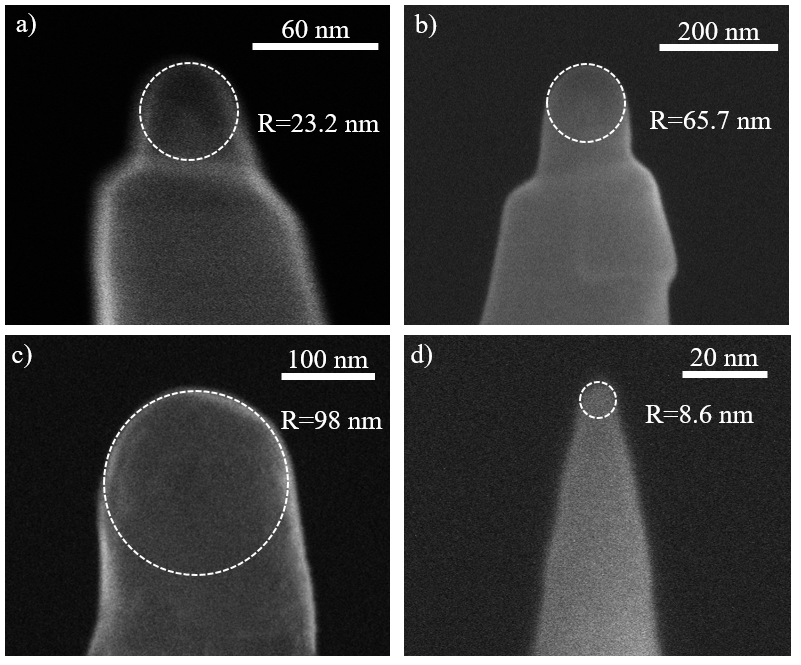


**FIG S3**. Post-measurement SEM characterization of the AFM probes used for data acquisition. (a-c) Micrographs of the silicon tips utilized for static force spectroscopy. The measured tip radii are R *≈* 23.2 nm (nominal 20 nm), 65.7 nm (nominal 60 nm), and 98 nm (nominal 100 nm), respectively. (d) Micrograph of the sharp silicon probe used for FM-AFM cross-validation, revealing a tip radius of R *≈* 8.6 nm. Dashed circles indicate the curvature fits used to determine the radii for the contact mechanics analysis.

**S2. FM-AFM VALIDATION AND DYNAMIC FORCE SPECTROSCOPY**

To provide a complementary consistency check of the adhesion values obtained via static force spectroscopy, we performed frequency-modulation AFM (FM-AFM) measurements [12]. This dynamic mode allows for the stable sampling of the conservative interaction potential without the "snap-in" instabilities inherent to static deflection methods, providing an additional check of the contact mechanics analysis. To recover the quantitative force-distance profile, $F_{ts}\left( z \right)$, from the measured frequency shift, *Δf(z)*, we employed the Sader-Jarvis inversion formalism [13]. The Sader-Jarvis equation reconstructs the force by integrating the frequency shift weighted by the oscillation amplitude:

$$F_{ts}\left( z \right)=\frac{2k}{f_{0}} \int_{z}^{\infty} \left[ \left( 1+\frac{A^{\frac{1}{2}}}{8\sqrt{\pi\left( t-z \right)}} \right)\Omega\left( t \right)- \frac{A^{\frac{3}{2}}}{8\sqrt{2\left( t-z \right)}} \frac{d\Omega\left( t \right)}{dt} \right]dt$$

(4)

where *k* is the cantilever stiffness, *Ω(t) ≡ Δf(t)*, and *t* is the integration variable for distance. Figure S4 summarizes the results of this dynamic analysis. The reconstructed force profiles (Fig. S4, right panels) clearly resolve the attractive van der Waals regime. To extract the clean-interface Hamaker constant, we fitted the attractive long-range tail of these profiles to the standard sphere-plane potential [14]:

$F_{vdW}\left( z \right)= -\frac{A_{H}R}{6z_{eff}^{2}}$ (5)

where R is the tip radius and *z_eff_*_​_ is the effective separation. Fig. S4 (bottom panel) shows the Hamaker constants derived from this dynamic reconstruction. These values lie within a 14% range with those obtained via the static method (see Fig. 2 in the main text), supporting the consistency of the interaction scale obtained by the two UHV approaches. This comparison should be regarded as a complementary check rather than a fully model-independent validation, since the FM-AFM analysis depends on force reconstruction, the fitting window, the effective tip geometry, and the definition of the absolute tip-sample separation.


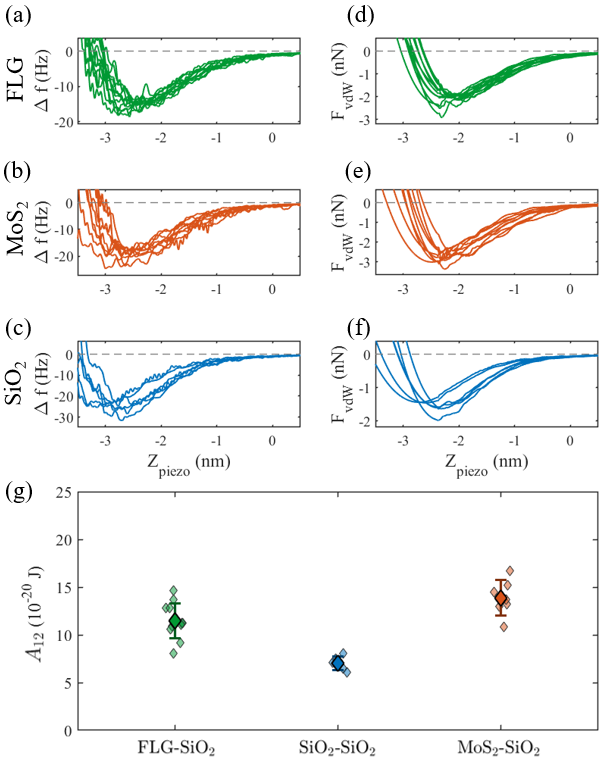


**FIG S4.** Complementary check of clean-interface adhesion via dynamic FM-AFM in UHV. (a, b, c) Frequency shift curves and corresponding reconstructed interaction forces (d, e, f) acquired on FLG (a, d), MoS_2_ (b, e), and SiO_2_ (c, f). (g) Hamaker constants (*A_12_*) extracted from the long-range tail fits. The error bars indicate the statistical dispersion of the acquired data.

**S3. CONTACT MECHANICS MODELS**

The adhesion force (*F_adh_*) was quantified experimentally as the magnitude of the "jump-off" instability in the retraction force curves (see Fig. 1 of the main text). To ensure statistical robustness and to identify possible geometric effects, we performed extensive force spectroscopy cycles across all material systems (FLG, MoS_2_, SiO_2_) using silicon probes with three distinct radii (*R ≈* 20, 60, 100 nm).

To interpret these measurements, we first estimated the contact mechanics regime using the Tabor parameter (*μ*), which describes the transition from the Johnson-Kendall-Roberts (JKR) to the Bradley/Derjaguin-Muller-Toporov (DMT) limits [15-17]:

$\mu= \left( \frac{16 R \gamma^{2}}{9 {K^{2}z}_{0}^{3}} \right)^{\frac{1}{3}}$ (1)

where R is the tip radius, *γ* is the work of adhesion, *K* is the reduced elastic modulus, and *z_0_* is the equilibrium separation. For silicon tips interacting with supported 2D crystals, we used *K* = 45 GPa as a conservative lower-bound reduced modulus for the silica-terminated tip/SiO₂-supported sample contact. For each material system, *μ* was calculated from the measured normalized adhesion force by first converting *F_adh_/R* into an effective Bradley work of adhesion, *γ = F_adh_/2πR* [18]. This calculation was performed for the *R =* 65.7 and 98 nm probes employed in the quantitative Hamaker extraction. Since
*μ ∝ K^−2/3^*, larger values of *K* would only decrease the estimated Tabor parameter. Table S1 summarizes the resulting upper-bound estimates. In all cases, *μ* remains below 0.3, placing the measurements on the low-Tabor, Bradley/rigid-body side of the JKR-DMT transition rather than in the JKR regime.

**Table S1.** Tabor parameter estimates calculated from the measured large-radius adhesion values. The effective Bradley work of adhesion is obtained from the normalized pull-off force as *γ = F_adh_/2πR*. Values were calculated using *K =* 45 GPa as a conservative lower-bound reduced modulus; larger *K* values would decrease *μ* as *K^−2/3^*^.^

| Material system | *z_0_* used for *μ* (nm) | *γ* (mJ m^−2^) | *μ, R* = 65.7 nm | *μ, R* = 98 nm |
| --- | --- | --- | --- | --- |
| FLG-SiO₂ | 0.3 | 35.4 | 0.14 | 0.16 |
| MoS₂-SiO₂ | 0.3 | 38.3 | 0.15 | 0.17 |
| SiO₂-SiO₂ | 0.2 | 40.5 | 0.23 | 0.26 |

Taking *z_0_ =* 0.3 nm as the more representative separation for FLG-SiO_2_ and MoS_2_-SiO_2_ contacts, the corresponding *μ* values remain below 0.17; even using *z_0_ =* 0.2 nm as a lower-bound distance increases them only to 0.24-0.25, still within the low-Tabor regime.

Consistent with these low-to-moderate Tabor parameters, we use the Bradley/Derjaguin rigid-body pull-off description [18-20]. In this low-deformation limit, adhesive necking is small and the pull-off force scales linearly with tip radius. Combining this pull-off relation with the Hamaker expression for the work of adhesion gives:

$F_{adh}=\frac{A_{H}R}{6 z_{0}^{2}}$ (2)

To evaluate this prediction, we normalized the measured adhesion forces by the tip radius (*F_adh_/R*) for all probes. As shown in Fig. 1(d) in the main text, this normalization groups the data for the larger-radius tips (*R ≈* 60 nm and *R ≈* 100 nm) onto a consistent value, supporting the linear scaling with radius expected for elastic contact mechanics in this regime. In contrast, the sharpest tips (*R ≈* 20 nm) exhibit higher normalized values, possibly due to the increased sensitivity of small-radius contacts to local stress, apex geometry, and radius-dependent or stiffness-dependent adhesive effects in thin supported systems [21-22]. The quantitative Hamaker constants are therefore extracted from the larger radius 60 and 100 nm probes, while the 20 nm data are retained as a small-radius control.

To make the statistical basis of the radius-scaling analysis explicit, Table S2 summarizes the UHV adhesion forces obtained for each material and SEM-measured tip radius. Values are reported as mean ± standard deviation over individual force curves.

**Table S2.** Statistical summary of the UHV adhesion forces used in the radius-scaling analysis. Values correspond to *F_adh_* and are reported as mean ± standard deviation. The SEM-measured radii are given in the column headers, allowing the corresponding *F_adh_/R* normalization to be directly obtained.

| Material | *F_adh_* (nN), *R =* 23.2 nm | *F_adh_* (nN), *R =* 65.7 nm | *F_adh_* (nN), *R =* 98.0 nm | No. of force curves / spatial locations |
| --- | --- | --- | --- | --- |
| FLG | 6.6 ± 0.3 | 15.0 ± 1.1 | 21.6 ± 1.0 | 113 / 9 |
| MoS_2_ | 8.5 ± 0.6 | 16.0 ± 1.3 | 23.8 ± 1.6 | 120 / 8 |
| SiO_2_ | 8.3 ± 0.7 | 16.8 ± 0.6 | 24.5 ± 2.0 | 138 / 9 |

The nominal 20, 60, and 100 nm tips correspond to SEM-measured radii of 23.2, 65.7, and 98.0 nm, respectively. The 20 nm tip was retained as a small-radius control, whereas the 60 and 100 nm tips were used for quantitative Hamaker extraction. The last column gives the total number of force curves and spatial locations used for each material across the three radii. One physical probe was used for each nominal radius and measured across the different material regions; therefore, independent tip-to-tip variability at fixed radius was not separately quantified.

Accordingly, we converted adhesion into Hamaker constants using the effective contact distances adopted and discussed in the main text: *z_0_* *=* 0.3 nm for FLG and MoS_2_, and *z_0_* *=* 0.2 nm for the SiO_2_-SiO_2_ reference. The values obtained with *z_0_* *=* 0.2 nm for FLG and MoS_2_ are also reported in the main text to illustrate the sensitivity of adhesion-based mappings to this assumption.

$A_{12}=\frac{6 F_{adh}z_{0}^{2}}{R}$ (3)

Because short-range repulsive interactions can influence pull-off-based Hamaker estimates, *z_0_* is kept explicit in Eq. (3) and the associated z_0_-dependent variation is treated as a systematic uncertainty [23].

While calculations were performed for all probes, we use the larger-radius tips (R *≈* 60 and 100 nm) as the clean-interface baseline for quantitative Hamaker constant extraction, with the 20 nm data serving as a small-radius control that illustrates the limits of the simple scaling regime.

**References**

[1] A. Castellanos-Gomez, M. Buscema, R. Molenaar, V. Singh, L. Janssen, H. S. J. van der Zant, G. A. Steele, “Deterministic transfer of two-dimensional materials by all-dry viscoelastic stamping,” 2D Materials, 2014, 1, 011002, https://doi.org/10.1088/2053-1583/1/1/011002.

[2] Z. Li, Y. Wang, A. Kozbial, G. Shenoy, F. Zhou, R. McGinley, P. Ireland, B. Morganstein, A. Kunkel, S. P. Surwade, L. Li, H. Liu, “Effect of airborne contaminants on the wettability of supported graphene and graphite,” Nature Materials, 2013, 12, 925-931, https://doi.org/10.1038/nmat3709.

[3] W. Bao, F. Miao, Z. Chen, H. Zhang, W. Jang, C. Dames, C. N. Lau, “Controlled ripple texturing of suspended graphene and ultrathin graphite membranes,” Nature Nanotechnology, 2009, 4, 562-566, https://doi.org/10.1038/nnano.2009.191.

[4] L. T. Zhuravlev, “The surface chemistry of amorphous silica. Zhuravlev model,” Colloids and Surfaces A: Physicochemical and Engineering Aspects, 2000, 173, 1-38, https://doi.org/10.1016/S0927-7757(00)00556-2.

[5] T. Takahagi, H. Sakaue, S. Shingubara, “Adsorbed Water on a Silicon Wafer Surface Exposed to Atmosphere,” Japanese Journal of Applied Physics, 2001, 40, 6198-6201, https://doi.org/10.1143/JJAP.40.6198.

[6] M. Donarelli, F. Bisti, F. Perrozzi, L. Ottaviano, “Tunable sulfur desorption in exfoliated MoS2 by means of thermal annealing in ultra-high vacuum,” Chemical Physics Letters, 2013, 588, 198-202, https://doi.org/10.1016/j.cplett.2013.10.034.

[7] Y. C. Lin, C. C. Lu, C. H. Yeh, C. H. Jin, K. Suenaga, P. W. Chiu, “Graphene annealing: how clean can it be?,” Nano Letters, 2012, 12, 414-419, https://doi.org/10.1021/nl203733r.

[8] I. Horcas, R. Fernández, J. M. Gómez-Rodríguez, J. Colchero, J. Gómez-Herrero, A. M. Baró, “WSXM: A software for scanning probe microscopy and a tool for nanotechnology,” Review of Scientific Instruments, 2007, 78, 013705, https://doi.org/10.1063/1.2432410.

[9] A. Gimeno, P. Ares, I. Horcas, A. Gil, J. M. Gómez-Rodríguez, J. Colchero, J. Gómez-Herrero, “‘Flatten plus’: a recent implementation in WSxM for biological research,” Bioinformatics, 2015, 31, 2918-2920, https://doi.org/10.1093/bioinformatics/btv278.

[10] L. Sirghi, O. Kylian, D. Gilliland, G. Ceccone, F. Rossi, “Cleaning and Hydrophilization of Atomic Force Microscopy Silicon Probes,” The Journal of Physical Chemistry B, 2006, 110, 25975-25981, https://doi.org/10.1021/jp063327g.

[11] J. E. Sader, J. W. M. Chon, P. Mulvaney, “Calibration of rectangular atomic force microscope cantilevers,” Review of Scientific Instruments, 1999, 70, 3967-3969, https://doi.org/10.1063/1.1150021.

[12] F. J. Giessibl, “Advances in atomic force microscopy,” Reviews of Modern Physics, 2003, 75, 949-983, https://doi.org/10.1103/RevModPhys.75.949.

[13] J. E. Sader, S. P. Jarvis, “Accurate formulas for interaction force and energy in frequency modulation force spectroscopy,” Applied Physics Letters, 2004, 84, 1801-1803, https://doi.org/10.1063/1.1667267.

[14] J. N. Israelachvili, Intermolecular and Surface Forces, 3rd ed., Academic Press, Burlington, MA, 2011.

[15] D. Maugis, “Adhesion of spheres: The JKR-DMT transition using a Dugdale model,” Journal of Colloid and Interface Science, 1992, 150, 243-269, https://doi.org/10.1016/0021-9797(92)90285-T.

[16] R. W. Carpick, D. F. Ogletree, M. Salmeron, “A general equation for fitting contact area and friction vs load measurements,” Journal of Colloid and Interface Science, 1999, 211, 395-400, https://doi.org/10.1006/jcis.1998.6027.

[17] D. Tabor, “Surface forces and surface interactions,” Journal of Colloid and Interface Science, 1977, 58, 2-13, https://doi.org/10.1016/0021-9797(77)90366-6.

[18] R. S. Bradley, “The cohesive force between solid surfaces and the surface energy of solids,” The London, Edinburgh, and Dublin Philosophical Magazine and Journal of Science, 1932, 13, 853-862, https://doi.org/10.1080/14786449209461990.

[19] B. V. Derjaguin, V. M. Muller, Y. P. Toporov, “Effect of contact deformations on the adhesion of particles,” Journal of Colloid and Interface Science, 1975, 53, 314-326, https://doi.org/10.1016/0021-9797(75)90018-1.

[20] J. A. Greenwood, “Derjaguin and the DMT Theory: A Farewell to DMT?,” Tribology Letters, 2022, 70, 61, https://doi.org/10.1007/s11249-022-01599-y.

[21] P. Ares, M. Pisarra, P. Segovia, C. Díaz, F. Martín, E. G. Michel, F. Zamora, C. Gómez-Navarro, J. Gómez-Herrero, “Tunable Graphene Electronics with Local Ultrahigh Pressure,” Advanced Functional Materials, 2019, 29, 1806715, https://doi.org/10.1002/adfm.201806715.

[22] C. Yu, W. Zeng, B. Wang, X. Cui, Z. Gao, J. Yin, L. Liu, X. Wei, Y. Wei, Z. Dai, “Stiffer Is Stickier: Adhesion in Elastic Nanofilms,” Nano Letters, 2025, 25, 1876-1882, https://doi.org/10.1021/acs.nanolett.4c05309.

[23] J. M. Vazquez, W. Oliver, S. P. Beaudoin, D. S. Corti, “The Effects of Short-Range Intermolecular Repulsive Forces on Hamaker Constant Estimation Using Atomic Force Microscopy,” Langmuir, 2024, 40, 24808-24819, https://doi.org/10.1021/acs.langmuir.4c02516.
